# Estimation of CpG coverage in whole methylome next-generation sequencing studies

**DOI:** 10.1186/1471-2105-14-50

**Published:** 2013-02-12

**Authors:** Edwin JCG van den Oord, Jozsef Bukszar, Gábor Rudolf, Srilaxmi Nerella, Joseph L McClay, Lin Y Xie, Karolina A Aberg

**Affiliations:** 1 Center for Biomarker Research and Personalized Medicine, School of Pharmacy, Virginia Commonwealth University, 1112 East Clay Street, P.O. Box 980533, Richmond, VA, 23298, USA

**Keywords:** Methylation, Next-generation sequencing, MBD/MeDIP, Association studies

## Abstract

**Background:**

Methylation studies are a promising complement to genetic studies of DNA sequence. However, detailed prior biological knowledge is typically lacking, so methylome-wide association studies (MWAS) will be critical to detect disease relevant sites. A cost-effective approach involves the next-generation sequencing (NGS) of single-end libraries created from samples that are enriched for methylated DNA fragments. A limitation of single-end libraries is that the fragment size distribution is not observed. This hampers several aspects of the data analysis such as the calculation of enrichment measures that are based on the number of fragments covering the CpGs.

**Results:**

We developed a non-parametric method that uses isolated CpGs to estimate sample-specific fragment size distributions from the empirical sequencing data. Through simulations we show that our method is highly accurate. While the traditional (extended) read count methods resulted in severely biased coverage estimates and introduces artificial inter-individual differences, through the use of the estimated fragment size distributions we could remove these biases almost entirely. Furthermore, we found correlations of 0.999 between coverage estimates obtained using fragment size distributions that were estimated with our method versus those that were “observed” in paired-end sequencing data.

**Conclusions:**

We propose a non-parametric method for estimating fragment size distributions that is highly precise and can improve the analysis of cost-effective MWAS studies that sequence single-end libraries created from samples that are enriched for methylated DNA fragments.

## Background

Methylation studies are a promising complement to genetic studies of variation in DNA sequence and structure. Most intensively studied is the methylation of DNA cytosine residues at the carbon 5 position (5^me^C). Methylation is typically associated with transcriptional repression [1,2]. This direct link to gene expression means that methylation studies can potentially capture more individual variation in disease susceptibility. Methylation studies can also shed a unique light on disease mechanisms and clinical phenomena [2,3] such as sex differences [4,5], genotype environment interactions [3,6], and age-related patterns associated with the disease course [7]. Finally, methylation sites are appealing from a translational perspective because they are modifiable by pharmacological interventions [8] and are easy to measure using cost-effective assays in readily available biosamples [9].

For most common, complex diseases, detailed prior biological knowledge is typically lacking. Therefore, genome-wide approaches that proved fruitful in the context of sequence variants [10] will also be critical to detect disease relevant methylation sites [11-13]. Next-generation sequencing (NGS) is an appealing technology for such methylome-wide association studies (MWAS). Compared to arrays, NGS provides better coverage of all possible methylation sites in the human genome [14]. Furthermore, the relatively low amounts of starting material will reduce errors and bias caused by sample preparation and amplification. Finally, the availability of fast semi-automated sample preparation, the increase in the amount of data generated per run, and the decrease in reagent costs have already made NGS a cost-effective option for a comprehensive interrogation of the methylome.

The most comprehensive method for ascertaining methylation (5^me^C) status at each nucleotide position is bisulfite sequencing [15], where unmethylated cytosines in genomic DNA are converted to uracil and then converted to thymine in post-bisulfite PCR [16]. The single base resolution is attractive because it allows precise mapping of disease relevant sites [14]. However, due to the combination of high costs of sequencing entire genomes and the large numbers of samples needed to provide adequate statistical power, whole-genome bisulfite sequencing is not currently economically feasible as a screening tool for disease association studies [11]. A commonly used cost-effective alternative aims to sequence only the methylated part of the genome. Here, DNA is first fragmented and the methylated fragments are bound to antibodies [17] or other proteins [18] with high affinity for methylated DNA. The unmethylated genomic fraction is washed away, and the methylation-enriched portion of the sample is then collected and sequenced [18-21].

Knowledge of the fragment size distributions in enrichment-based MWAS is important for several aspects of the data analysis. A clear example involves the calculation of enrichment measures. DNA methylation is most often, although not exclusively, found in the sequence context CpG. Certain enrichment protocols (e.g. MBD-based capture that uses the methyl binding domain of methyl binding proteins [18]), can even only detect CpG methylation. Given that we know exactly where the CpGs are located, there is no need to search for enrichment peaks using methods commonly used in ChIP-seq experiments [22-26]. Although there are other ways to quantify enrichment [27], a commonly used approach is to count the number of fragments covering each CpG. If the fragment sizes are unknown, the number of reads covering the CpG is typically counted instead [21], where read length is sometimes extended to the expected fragment length. However, this is a rough approximation. First, due to the stochastic nature of DNA fragmentation, one cannot assume an equal size for all fragments. Second, the expected fragment size may be mis-specified if the sequenced fragment pool differs from the one obtained after fragmentation. This could arise, for example, if smaller fragments are more likely to be pulled down in the enrichment step. Third, there will be variation in the fragment size distribution across samples despite standardized lab protocols. The possible implication of using the read count approximation is therefore that coverage estimates may become biased and imprecise.

A second example illustrating the importance of the fragment size distribution is that failure to account for differences in fragment size distributions between samples may create artificial inter-individual differences in coverage estimates. To illustrate this, assume that in sample A all DNA fragments are exactly 50 bases in length and that we sequence at 50 bp read length. When using a read count to calculate coverage for the CpG that caused the enrichment, all reads will contribute to this count because the start positions of all aligned reads will be within 50 bp of that methylated CpG. Now assume a second sample B that has identical methylation levels at the target CpG. However, for this sample all fragments are 200 bp long, but we still sequence only 50 bp of each fragment. As only a proportion of the reads would now start within 50 bases of the CpG, the read count will be less than for sample A and underestimate the number of fragments covering the target CpG.

Rather than using an estimation procedure, by sequencing paired-end libraries we can obtain the fragment size distribution by subtracting the start positions of successfully aligned read pairs. However, paired-end libraries have only recently become available, so legacy data is typically single-end and not all sequencing platforms currently support paired-end libraries. Second, the use of paired-end libraries is more expensive and almost doubles the sequencing run time. These disadvantages may be justified in studies that require single base resolution, such as calling DNA sequence variants or estimating the percentage of methylation at specific bases after bisulfite conversion. In these scenarios, the number of reads covering each base is a critical determinant of data quality. However, for the enrichment-based methylation studies considered in this paper, it is the number of sequenced fragments that determine data quality. As the use of paired-end libraries does not increase the number of fragments, one could argue that it is better to spend the additional resources on sequencing more fragments using single-end libraries.

The goal of our investigation is to develop a method that uses single-end sequencing data to estimate fragment size distributions. Due to the nature of the sequencing technology as well as specific lab procedures to optimize the assays (e.g. size selection on fragments prior to sequencing), it is difficult to make strong parametric assumptions about this distribution. Therefore, we propose a non-parametric method. To validate our method, we performed simulation studies and made comparisons with NGS studies of paired-end libraries, which provide a benchmark by allowing an empirical determination of the fragment size distribution.

## Methods

A detailed exposition of the proposed method to estimate CpG coverage can be found in the supplemental material and we confine ourselves here to a summary. In contrast to for example Chip-seq data, in methylation studies there are often many sites that are located close to each and all can affect the enrichment. This complicates the estimation of the fragment size distribution. For example, using all reads in the neighborhood of a CpG that has other CpGs nearby will give imprecise estimates of the fragment size distribution because part of the enrichment at that locus will be the result of the nearby CpGs. To address this problem, our method uses only isolated CpGs. An isolated CpG is defined as a site C for which the interval [C-*d*,C + *d*] contains no other CpGs but C and where *d* is larger than the longest possible fragment size. For these isolated CpGs it is reasonable to assume that, given the fragment size *X* = *x*, the possible nucleotide positions *R* where the reads can start is independent and uniform distributed (see Additional file 1: Figure S3 and Results section for empirical support of this assumption).

From this assumption it follows that the probability mass function of the possible read start position *R* equals:

(1) $$\mathrm{Pr}\left(R=r\right)=\sum_{x=r+1}^{s}\frac{\mathrm{Pr}\left(x\right)}{x}$$

where Pr(*x*) denotes the probability that fragments have size *x*, and *s* is the size of the longest fragment. The summation over fragments starts at *r* + 1, because fragments can only have reads with a start position *r* if *x* > *r*. We obtain the probability mass fragment size function by solving Pr(*x*) from (1):

(2) $$\mathrm{Pr}\left(X=r\right)=\left(\mathrm{Pr}\left(R=r-1\right)-\mathrm{Pr}\left(R=r\right)\right)\times r$$

Because the enrichment is imperfect, not all sequenced fragments will contain methylated CpGs. Under the assumption that the start positions of such “noise” reads are uniformly distributed in the [C-*d*,C + *d*] interval, they will not bias estimates because Pr(x) in (2) is calculated from the difference between the numbers of reads starting at adjacent bases.

We need to know for each read the probability that the fragment it is tagging covers that CpG. This coverage function is equal to the complement of cumulative fragment size function 1-(F(*x*)) or

(3) $$\mathrm{Pr}\left(X>r\right)=\sum_{x=r+1}^{s}\mathrm{Pr}\left(x\right)$$

For example, if the read length is 50, Pr(X > r) = 1.0 for reads starting within 50 bp of the CpG but Pr(X > r) < 1.0 for reads starting further away as part of the tagged fragments will be too short to cover the CpG. Once we determined for every read the probability that the fragments they are tagging cover the CpG, these probabilities can be summed over all reads to obtain a coverage estimate for the CpG.

An expected contribution of a randomly chosen read to the coverage, E(cov), can be calculated by combining (1) that gives the distribution of read start positions with (3) that specifies how reads starting at these locations contribute to coverage:

(4) $$E\left(\mathrm{cov}\right)=\sum_{x=0}^{s-1}\mathrm{Pr}\left(R=r\right)\mathrm{Pr}\left(X>r\right)$$

Equation (4) shows that E(cov) depends on the fragment sizes. The implication is that coverage estimates will differ across samples if the fragment sizes differ across these samples. However, because the fragment size distribution is determined by the lab protocol and is not directly related to the amount of methylation, this difference represents an artifact. To avoid such differences it may be necessary to standardize the coverage estimates using this expected contribution. We calculated the required coverage standardization factor for each sample as the mean of the expected read contributions across all samples in the study divided by E(cov) in (4) for that specific sample.

### Estimation procedure

We apply the following stepwise procedure to estimate the coverage function in (3).

**Figure 1:**
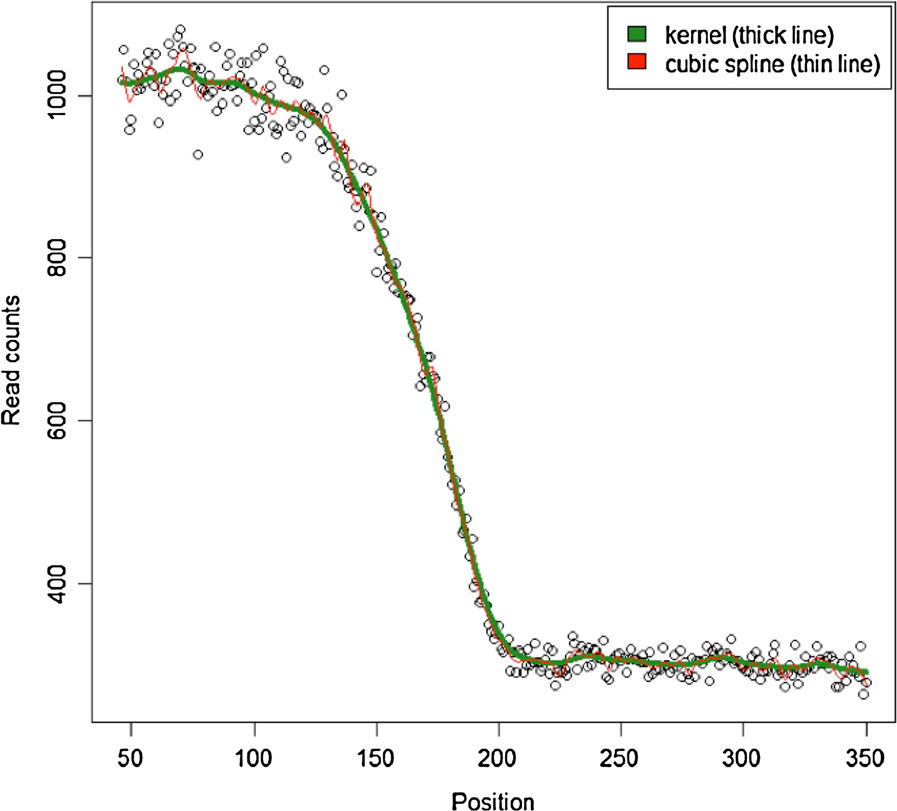
**Counts of read start positions and fitted smoothing functions.** Points are actual counts, thick line is the kernel-based smoother, and the thin line is the qubic spline smoother.

1) Select the isolated CpG sites for the chosen interval [C-*d*,C + *d*] and count all the read start positions in the vicinity of these isolated sites. For reads aligning to the forward strand this involves all reads starting in the C-*d* interval that is upstream of the CpG, and for reads on the reverse strand all reads starting in the (C + 1) + *d* interval that is downstream of the CpG. A value for *d* can be obtained from a visual inspection of an initial plot of the read start counts. For example, the dots in Figure 1 show an example where these counts decrease until position 240 after which they start to fluctuate around the “noise” level. This pattern suggests that very few fragments are longer than 240 and for *d* we could therefore choose a value between 250–300 bp.

2) As the read start counts will show sampling fluctuations, we “smooth” the data prior to calculating Pr(*x*) with formula (2). Our first method involved the Nadaraya-Watson estimator [28,29]. This smoother takes for each position the *m* nearest neighbors and estimates the number of read starts by averaging the values across this window using a kernel as a weighting function. As it essentially uses the mean, this kernel-based smoother assumes that the underlying function is locally constant. To provide an alternative we also used a cubic spline method that fits a more flexible local regression model instead. We illustrate results obtained with the two methods in Figure 1.

3) Step 2 results in a set of estimates of Pr(*x*) that are used in step 3 to calculate the coverage function (3). The true underlying coverage function (3) is monotone descending but in practice this may not hold due to sampling fluctuations. To ensure monotonicity, as a final smoothing step we used the procedure proposed by Dette et al. [30,31].

To obtain the coverage functions and standardization factors we wrote an R function that also summarizes and plots the (smoothed) data. For the kernel-based smoother we used the R function ksmooth. For the cubic spline we used smooth.spline, the design of which parallels the smooth.spline function of Chambers and Hastie [32]. The function monoProc was used in the final step to obtain monotone descending coverage functions. We also created a program to create the input data, which is a table with the counts of the read starts around isolated CpGs. Prior to calculating this table, the program allows user specified quality control (QC) of the reads. Because of the size of the data files, this program was coded in C++. The source code, Windows and Linux executables, and documentation are freely available from http://www.people.vcu.edu/~ejvandenoord/.

### Empirical data used to test method

To validate our method we sequenced 50 bp + 35 bp paired-end libraries in 8 inbred adult C57BL/6 male mice (see supplemental material for details on the laboratory methods, quality control, and data processing). The number of reads per sample was on average 53.6 million. We could map 87% of the reads. Using *d* = 350 bp, the total number of isolated CpGs was 287,493 which corresponds to 1.4% of all CpGs in the C57BL/6 genome (build 9/NCBI37). In terms of uniquely mapped reads, an average of 184,853 reads per sample mapped to isolated CpGs.

Fifty-two percent of the mapped reads (or 45% of total reads) satisfied our criteria for high quality read pairs meaning that they aligned uniquely with the right orientation and acceptable fragment size. We obtained the fragment size distributions from the paired-end data by subtracting the start positions of the successfully aligned read pairs. Although these fragment size distributions may not be perfect (e.g. only a proportion of the read pairs are used and the fragments tagged by these high quality pairs may not be completely representative of all sequenced fragments), they should provide a good opportunity to validate findings as the distributions are “observed” and do not require estimation.

## Results

### Simulation studies

**Figure 2:**
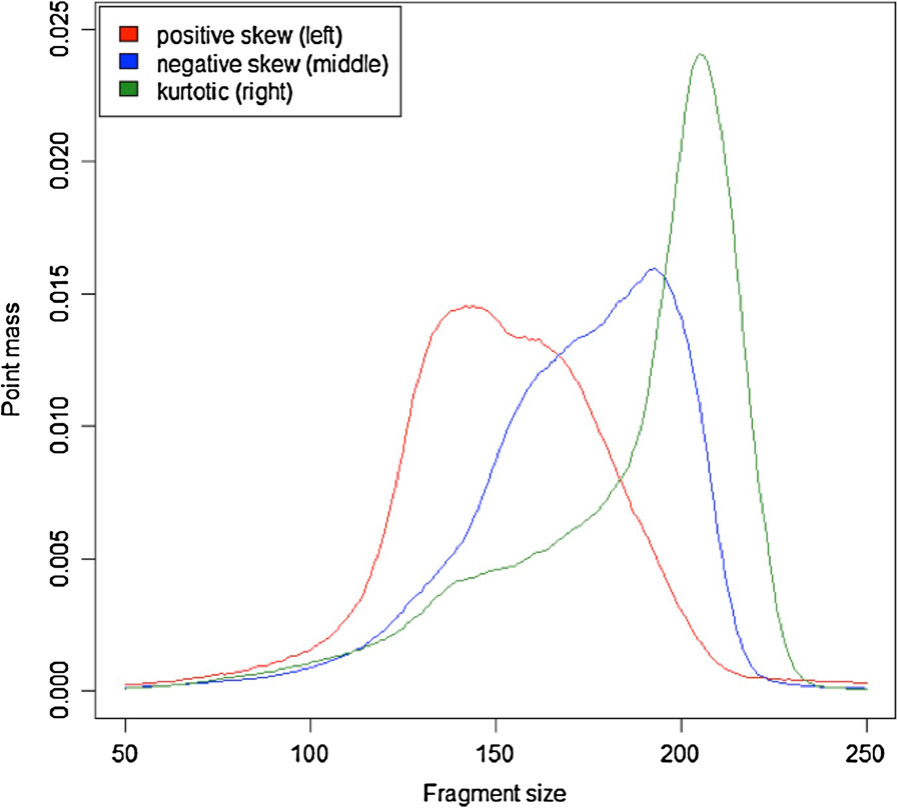
**Probability mass fragment size distributions.** The three examples were obtained using successfully aligned read pairs our sequencing study in mice.

Figure 2 depicts three fragment size probability mass functions, selected from our sequencing study in eight mice, which were calculated from the paired-end data as indicated above. The figure clearly shows non-normal distributions supporting our argument that non-parametric methods are needed to accurately characterize them. Part of the non-normality can be explained by size selection, a standard step in NGS library construction that aims at eliminating short and long fragments. The sharp decline in the number of fragments at around 230–240 bp seen for the kurtotic distribution, for example, is likely the result of successfully eliminating long fragments. Figure 2 also shows that despite the use of size selection and a standard protocol to fragment the DNA, considerable variation exists in the individual fragment size distributions. This suggests that fragment size estimation methods assuming the same distributional form for all samples are at risk for producing biased results.

To test our estimation procedure through simulations, we generated 10,000 random samples based on each of the three distributions in Figure 2. The number of reads with start positions close to isolated CpGs equaled 10,000, 25,000, 50,000, 75,000, or 100,000. The condition that assumes 100,000 reads is comparable to what we observe in our empirical data. The other conditions enable us to get a sense of the robustness of our method in case fewer reads would be available. To assess the precision of our estimator, we first calculated the mean difference and absolute mean difference between the estimated coverage function and the real coverage function used to simulate the data, and then averaged these differences across all possible read start positions. When subsequently averaged across the 10,000 simulated samples, the mean difference provides information about whether there are systematic differences (=bias) between estimated and true coverage functions. To obtain a measure of the variability of the estimated coverage functions, we also calculated the standard deviation of the mean difference in the 10,000 simulations. Finally, the mean of the absolute differences across the 10,000 simulations provides an overall measure of precision that incorporates both systematic differences and the variability of the estimates.

All three statistics in Table 1 show that precision increased with sample size. The mean was very close to zero, suggesting that the estimates were unbiased. The small standard deviation and mean absolute difference suggested that our method was precise. In addition to sample size, the fragment size distribution type affected the precision of the estimates (see Additional file 1: Figure S1). The least precise estimates were obtained for the kurtotic distribution and the most precise estimates for the distribution that was positively skewed. In Table 1 results for the kernel-based method are shown but those obtained using cubic splines were almost identical.

**Table 1:** Summary of simulation results comparing estimated and true coverage curves with different numbers of reads

**# reads**	**10,000**	**25,000**	**50,000**	**75,000**	**100,000**
Mean	−0.00017	−0.00018	−0.00024	−0.00034	−0.00060
Standard deviation	0.00693	0.00444	0.00323	0.00263	0.00212
Absolute difference	0.01225	0.00827	0.00613	0.00512	0.00461

**Figure 3:**
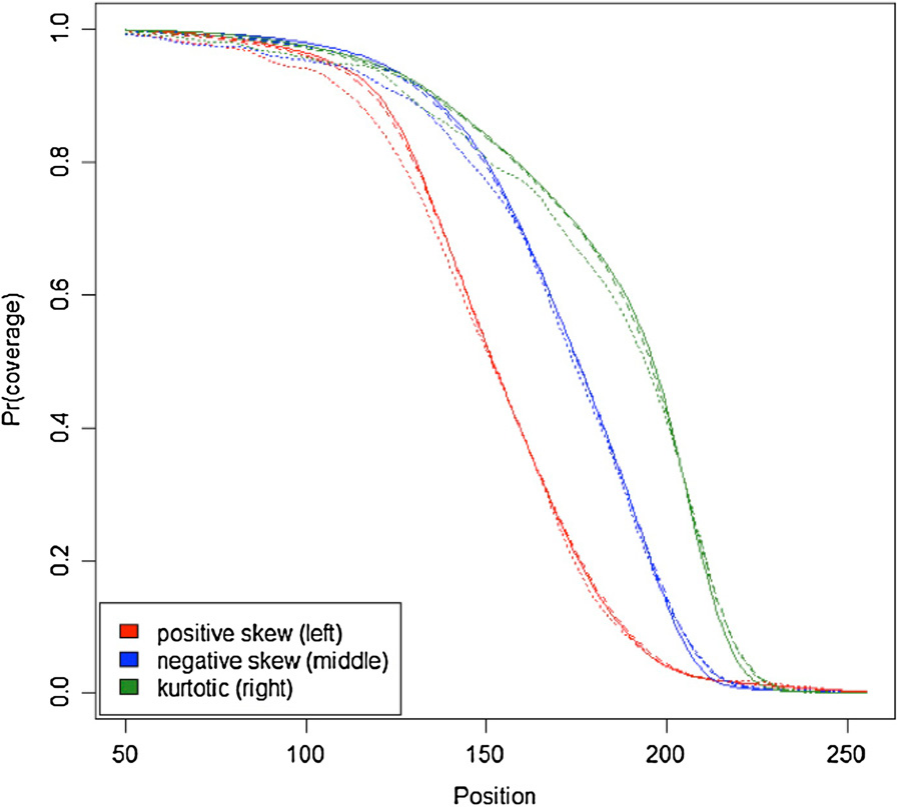
**Selection of estimated coverage functions for simulation conditions with 50,000 reads.** The three fragment size distributions depicted in Figure 2 were used to simulate the data, where the coverage function implied by these three distributions is represented by the solid lines. The dashed lines indicate the mean of the estimated across all 10,000 simulations. The dotted line is an example of an estimated coverage function that has mean error identical to the 99th percentile of the 10,000 estimates.

Figure 3 displays estimated coverage functions for the condition with 50,000 reads. The Additional file 1: Figure S2 a-d shows similar plots for the conditions with 10,000, 25,000, 75,000, and 100,000 reads. Results are shown for the three fragment size distributions depicted in Figure 2 that were used to simulate the data, where the coverage function implied by these three distributions is depicted as well. Figure 3 shows that the mean of the estimated coverage functions across all 10,000 simulations almost perfectly traced the coverage function used to generate the data. This suggests that our estimation procedure is unbiased. The figure also displays an example of a coverage function that has a mean absolute difference identical to the 99th percentile of all 10,000 estimates. The fact that this curve is also very close to the true coverage function suggests that the variability is modest and that our method almost always yields a good approximation to the actual function. Further analyses showed that our method is robust, because even with as few as 10,000 reads (Additional file 1: Figure S2a) with start positions around isolated CpGs, the estimation is precise.

### Estimating coverage functions with empirical data

Additional file 1: Figure S3 shows the plots with read start position distributions for each of the 8 samples. These distributions show systematic outliers at the very beginning of the read (positions 0–4). However, after these initial positions, the frequencies of the read start positions do not show a systematic trend until the read length is reached. The decay after that point is expected and caused by parts of fragments becoming too short to cover the CpG (see Formula 1), which essentially forms the basis of our estimator. In other data, we have sometimes observed a decay that starts before the read length is reached. However, this was the result of some fragments being shorter than the read length. Such fragments can occur when the instrument initially sequences part of the adaptor. These reads are then “trimmed” during alignment. Thus, when the methylated CpG is at the very beginning of the read, the assumption of uniform read start distribution does not hold. However, as our estimator only uses the data starting from approximately the minimum read length, these outliers do not affect the estimation. The absence of a systematic trend until the minimum fragment length is reached suggests that the assumption of a uniform distribution for position-level read counts is reasonable for the range from which the data are used.

Before estimating the coverage function using the empirical sequencing data, we first eliminated one read from each pair as to create single-end read input data. Empirical data will comprise multi- and duplicate-reads. Many reads map to multiple locations of the genome. Often a single alignment can be selected because it is clearly better than the others. In the case of multi-reads, multiple alignments are about equally good. Selecting only the best alignment for each multi-reads read carries along the danger of alignment errors (e.g. alignments to regions with SNPs are less likely to be best alignments because SNPs cause mismatches). On the other hand, excluding all multireads may affect accuracy in a negative way [33]. Duplicate-reads are reads that start at the same nucleotide positions. When sequencing a whole genome duplicate-reads often arise from template preparation or amplification artifacts. In our context of sequencing an enriched genomic fraction, duplicate-reads are increasingly likely to occur by chance because reads are expected to align to a much smaller fraction of the genome.

We examined empirically whether it would be better to allow for a limited set of high quality multi- and duplicate reads or exclude all such reads. To select high quality multi-reads, any read that mapped to more than 10 loci was excluded from further consideration. From the remaining multi-reads, we selected those that aligned almost equally well to only a few loci. Specifically, we selected the multi-reads that had fewer than five alignments with alignment scores (read length ‒ 3 × the number of mismatches) within five points of the best score. To avoid disproportionate representation, multi-reads were weighted in proportion to the number of alignments in the coverage calculations. In all instances where >3 (duplicate) reads started at the same position, we reset the read count to 1 for the coverage calculations assuming that these reads all tagged a single fragment. If 2 or 3 reads started at the same position, we looked for other reads in neighborhood ±25 bp. If other reads mapped to this area, we retained the read count of 2 or 3 in the coverage calculations, assuming that the duplicate reads occurred by chance due to enrichment of fragments caused by methylated CpG in the region. If no other reads were found, we assumed that the duplicate reads were artifacts and reset the read count to 1 for the coverage calculations.

In Table 2 we report the mean, standard deviation, and absolute mean difference between the estimated coverage function (see text Table 1 for discussion of these indices) and the coverage function as implied by the paired-end fragment size distributions of the 8 samples. The most precise results were obtained by including high-quality multi- and duplicate-reads. Here, the mean was closest to zero indicating almost unbiased estimates, the standard deviation was smallest implying that the estimates were less variable, and the mean of the absolute difference that is a function of both a possible bias plus the variability in the estimates was also smallest. Comparisons suggest multi-reads are more critical for precision than duplicate-reads. As shown by the first row of Table 2, the proportion of high quality multi- (about 20%) and duplicate reads (about 15%) can be substantial. This larger number of observations when multi- and duplicate- reads are used in for the estimation may explain the higher precision of the estimates.

**Table 2:** Precision coverage function estimated after different QC procedures for duplicate- and multi-reads

	**multi**	**multi**	**no multi**	**no multi**
	**duplicate**	**no duplicate**	**duplicate**	**no duplicate**
# reads/sample	297,405	253,601	217,745	184,853
Mean	−0.0078	−0.0079	−0.0112	−0.0132
Standard deviation	0.0214	0.0217	0.0222	0.0243
Absolute difference	0.0178	0.0181	0.0194	0.0207

Note: # reads/sample is the number of reads around isolated CpGs used as input for our estimation method.

**Figure 4:**
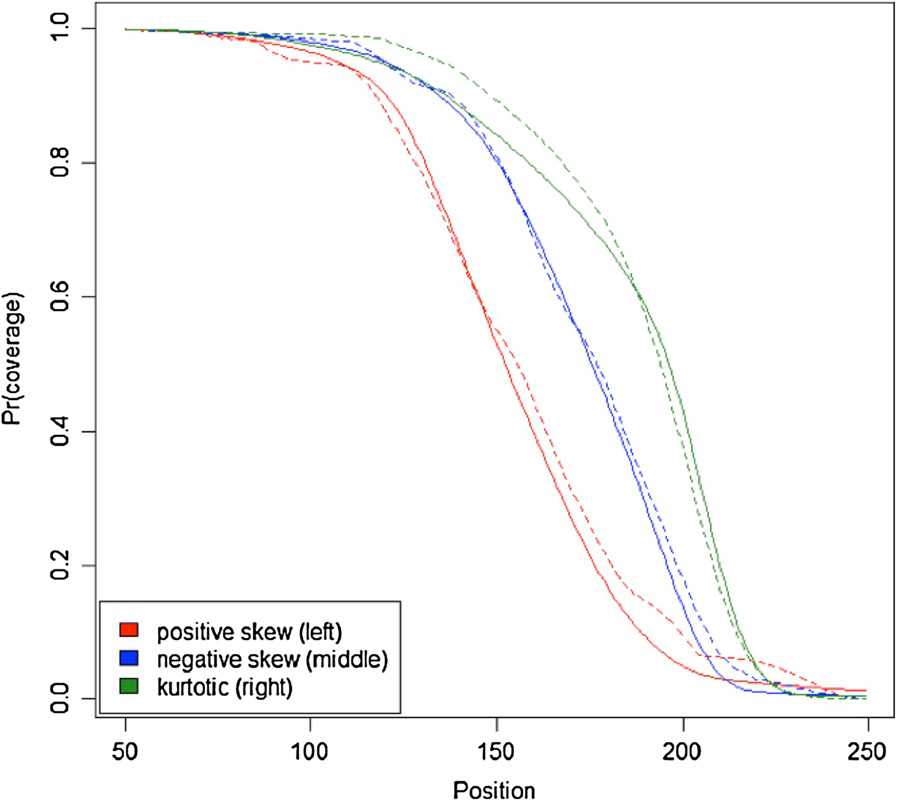
**Coverage functions estimated from the empirical data.** The solid lines represent three distributions calculated using successfully aligned paired-end reads. The dashed lines are the estimates that allow for high quality multi- and duplicate reads.

**Figure 5:**
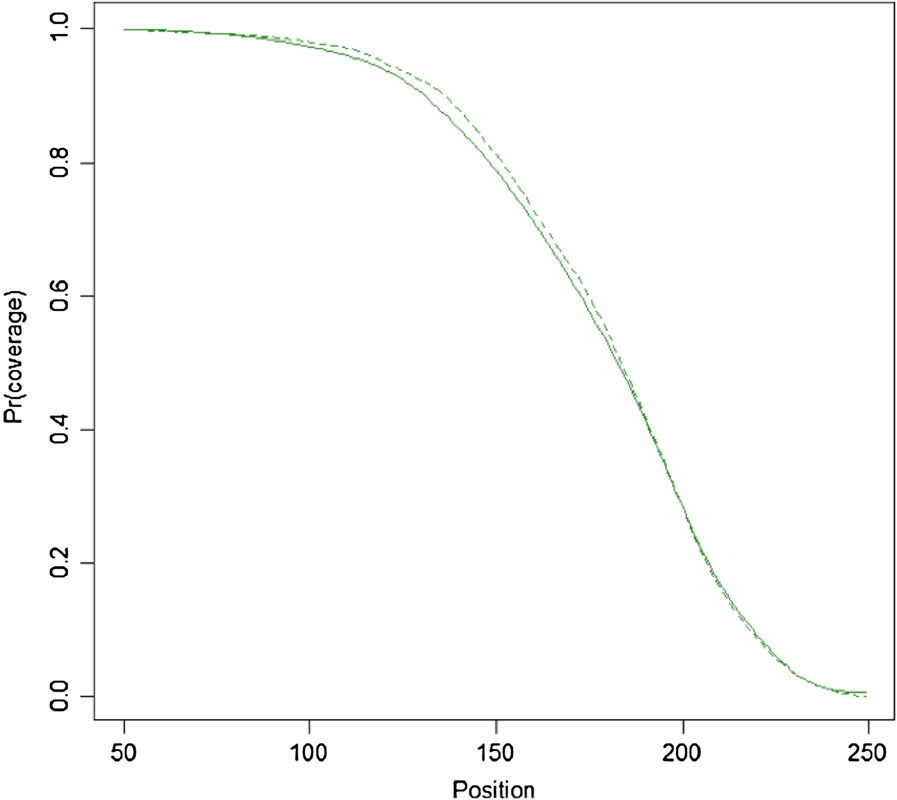
**Mean coverage function for all 8 samples.** The solid lines represent three distributions calculated using successfully aligned paired-end reads. The dashed lines is an estimate based on the mean across all 8 samples.

Figure 4 displays the coverage functions obtained using the successfully aligned paired-end read pairs from the three samples from Figure 2 as well as the estimated functions. The good correspondence suggested that the estimation method worked well with empirical data. However, considering the relatively large number of reads, the correspondence is not as good as observed with the simulated data. This may be because of assay related factors that influence empirical read start data and/or the possibility that paired-end data may provide only an approximation of the true coverage function. In Figure 5 we show the mean of the estimated coverage functions across all eight mouse samples. This mean closely tracks the corresponding mean in the paired-end data suggesting that most of the deviations seen for the individual estimates in Figure 4 are not systematic. As the use of the mean distribution may be an alternative in situations where the individual distributions are considered unstable, we will study this mean function in our next section on coverage estimation as well.

### Coverage estimation

In Table 3 we report results from various coverage calculations performed on the mouse data. Coverage was calculated for all 20.4 million CpGs of the 19 autosomal mouse chromosomes. The first row shows the coverage calculations using the fragment size distributions as “observed” in paired-end read data. These coverage estimates were used as the benchmark. Next, we present a “traditional” coverage calculation where we counted the number of sequence reads covering the CpGs. Results show that this method severely underestimates the coverage. More precisely, the “ratio” column shows that the mean coverage is merely 29.2 percent of that obtained after analyzing the paired-end data. Furthermore, when we correlated these coverage estimates with those obtained from the paired-end data, we only obtained a very modest correlation of 0.606. The DNA samples were fragmented by ultrasonication (Covaris, Woburn, MA) to a target median size of 150 bp. For the coverage calculations in the next row of Table 3 we extended the read length from 50 bp to this 150 bp target. Results improved but coverage was still underestimated by 13% and the correlation with paired-end coverage estimates was 0.934. In the row labeled “kernel estimate”, we used our method to estimate individual coverage functions for all 8 mice thereby including high quality multi- and duplicate reads. Results were now very similar to the results obtained with the paired-end data with only a slight overestimation of 0.6 percent. In addition, these coverage estimates correlated 0.999 with the estimates from the paired-end data suggesting almost identical results. We also explored whether using the mean coverage function produced even more precise results. This was not the case. The most likely explanation is the use of a mean function for estimating coverage when in reality considerable individual differences in fragment size distributions exist.

**Table 3:** Comparison of coverage estimates obtained using different methods

	**Mean**	**SD**	**Ratio**	**Correlation**
Paired-end	6.168	3.282	100.0%	1.000
Read count (50 bp)	1.824	1.357	29.6%	0.608
Read count extended (150 bp)	5.385	3.036	87.3%	0.934
Individual kernel function	6.207	3.287	100.6%	0.999
Mean paired-end function	6.202	3.331	100.6%	0.986
Mean kernel function	6.251	3.356	101.4%	0.986

Note: Ratio is mean divided by mean of paired-end data and Correlation is the correlation between coverage calculations with the method listed in the row versus those obtained from the paired-end data.

**Figure 6:**
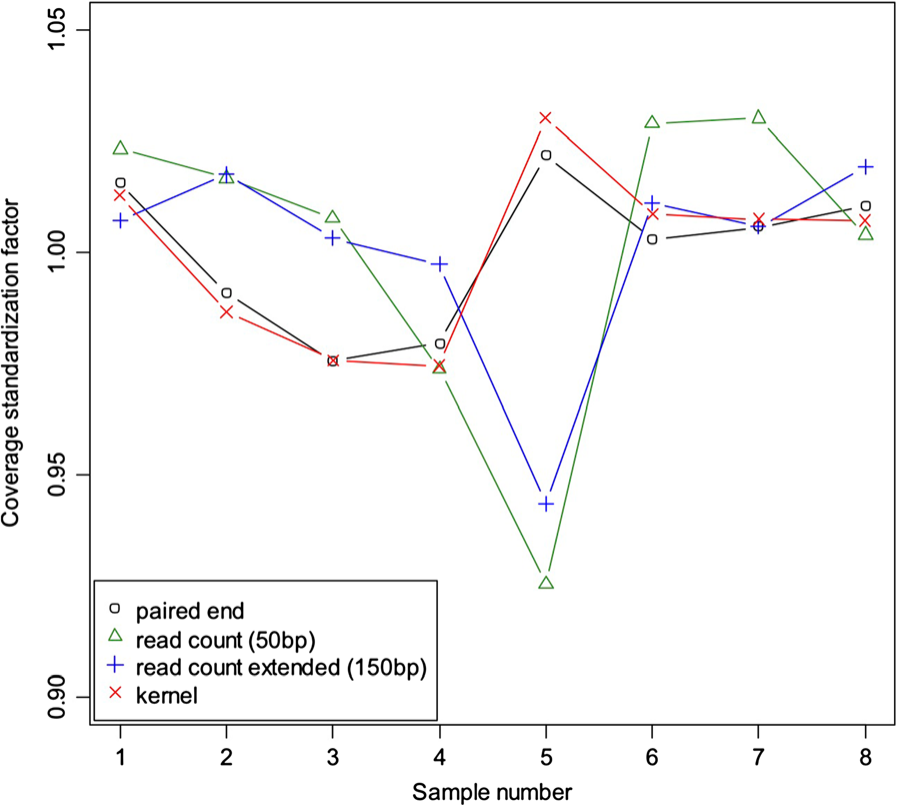
**Coverage standardization factors.** The ○ represent the standardization factors calculated using the fragment size distribution obtained from the paired-end data. The ∆ represent coverage standardization factors that would be needed for the read count method and the + for the extended read count method, where in both cases we used the fragment size distribution obtained from the paired-end data for the calculations. The × sign represent estimates of the standardization factors for which we used the kernel smoother.

Figure 6 shows the coverage standardization factors. The results indicate that individual variation exists in coverage standardization factors. Therefore, if coverage is not corrected, it will differ across samples in proportion to the standardization factors. The issue is that these differences are not the result of methylation differences but (arbitrary) differences in fragment size distributions between samples. Although the bias will be in the opposite direction, Figure 4 shows that this also occurs for the traditional (extended) read count method. Thus, regardless of the way coverage is calculated, a standardization step based on the fragment size distributions is needed to avoid biased results. The figure also shows the correspondence between the coverage standardization factors from the paired-end read data and coverage calculated after estimating the fragment size distributions. This suggests that our method can be used for the purpose of standardizing coverage as well.

## Discussion

We developed a non-parametric method that uses isolated CpGs to estimate sample specific fragment size distributions from data obtained by sequencing single-end libraries. An important application of the proposed method is to quantify the amount of methylation by estimating the number of fragments covering a CpG. To optimize coverage estimation, we studied several variations. Although it is possible that the optimal approach varies somewhat across settings, we found that the two smoothing methods had very similar overall performance. Furthermore, the inclusion of particularly high quality multi-reads, rather than merely using uniquely mapped reads, improved the precision of the estimated coverage function. This finding is consistent with other reports showing that multi-reads contain information and should not automatically be discarded [33]. Finally, the use of a mean coverage function across all samples may result in a loss of precision. This is because the reduced sampling fluctuations may not outweigh the biases that are introduced when a mean function is used to approximate fragment size distributions that are likely to vary across samples, even if stringent lab protocols are used to minimize these differences.

Our data suggested that taking the fragment size distribution into account may be important to obtain unbiased coverage estimates even when the standard (extended) read count method is used for coverage calculations. Thus, using the mouse sequence data, we showed that even after careful size selection and the use of a standardized protocol to fragment DNA, differences in fragment size distributions can occur that can create artificial inter-individual differences in coverage estimates. To avoid these biases we proposed a standardization factor that can be calculated from the estimated fragment size distributions.

Further applications of the estimated fragment size distributions are conceivable as well. For example, enrichment-based methods are semi-quantitative in the sense that they do not yield direct estimates of methylation levels. For the purpose of assessing methylation levels of sites, methods have been developed to remedy this problem by normalizing the data based on local CpG density [34,35]. However, the optimal definition of CpG density depends on the fragment size distribution. For example, the local CpG density of a site will be higher if the fragments are larger. Thus, the proposed method can yield a more refined measure of CpG density.

Our estimator uses data from isolated CpG sites, which correspond to a modest proportion of all CpGs (e.g. 1.4% in C57BL/6 mice). It is possible that fragment length distribution differs for the remaining CpG sites. It is important to note that such a bias would affect our method but not the coverage function derived from the paired-end sequencing data that considers all fragments. The fact that we observed a correlation of .999 between coverage calculations based on our estimate versus those based on the paired-end coverage function suggests that a possible bias does not interfere with the precision of our method. A somewhat related point is that enrichment protocols may be less efficient for CpG poor versus CpG rich regions [21], and that the enrichment will depend on the extent isolated CpGs are methylated. As the precision of our method depends on the successful enrichment of fragments with a single methylated CpG, it may not work as well with protocols that mainly enrich for CpG dense regions or in samples where isolated CpGs are not methylated.

## Conclusions

Methylation studies are a promising complement to genetic studies of DNA sequence. However, detailed prior biological knowledge is typically lacking, so methylome-wide association studies will be critical to detect disease relevant sites. A cost-effective approach involves sequencing single-end libraries created from samples that are enriched for methylated DNA fragments. A limitation of single-end libraries is that the fragment size distribution is not observed, which hampers several aspects of the data analysis. In this article we developed a non-parametric method that uses isolated CpGs to estimate sample specific fragment size distributions. We show that our method is highly accurate and can improve the analysis of cost-effective MWAS studies that sequence single-end libraries created from samples that are enriched for methylated DNA fragments.

## Abbreviations

NGS: Next-generation sequencing; MWAS: Methylome-wide association studies; QC: Quality control.

## Competing interest

The authors report no conflicts of interest.

## Authors’ contributions

GR, JB, and EVDO and developed the method. LYX generated the data used to validate the method. KA and JLM contributed theoretical expertise about sequencing technology and methylation. EVDO implemented the method. EVDO and SN the performed data analyses. EVDO, JLM and KA drafted the MS. All authors read and approved the final manuscript.

## Supplementary Material

Additional file 1 Supplemental material for the paper: Estimation of CpG coverage in whole methylome nextgeneration sequencing studies. Click here for file
