# Supplementary material for: Estimation of CpG coverage in whole methylome next-generation sequencing studies
Source: BMC Bioinformatics. 2013 Feb 12;14:50. doi: 10.1186/1471-2105-14-50 (PMC3599116; doi:10.1186/1471-2105-14-50)
Supplement: Additional file 1 — Supplemental material for the paper: Estimation of CpG coverage in whole methylome nextgeneration sequencing studies. [file 1471-2105-14-50-S1.pdf]

Supplemental material for the paper:

Estimation of CpG coverage in whole methylome next-generation sequencing studies

Contents

1. Estimating the coverage function from position-level read counts 2

2. Study samples 3

3. DNA methylation profiling.. 3

4. Simulation results... 4

Figure S1. Simulation results for curve type 4

Figure S2: Estimated coverage functions with 10k, 25k, 75k and 100k reads 5

5. Plots of counts for read start positions... 6

Figure S3. Counts of read start positions for each of the 8 samples. 6

1. Estimating the coverage function from position-level read counts

Assume a uniform distribution for the read start positions $R = r$ given fragment size $X = x$

$$f(R = r|X = x) = \left\{ \begin{array}{c} \frac{1}{x}\text{for } 0 \le r < x \\ 0 \text{ for } r \ge x \end{array} \right\}$$

If $Pr(x)$ denotes the probability that fragments have size x, s_0 is the size of the shortest fragment we can sequence, and s is size of the largest fragment, then the probability of reads starting at location r is

$$\Pr(R = r) = \sum_{x=s_0}^{s} Pr(x) f(R = r|X = x)$$

Because fragments of size x can only have reads starting at r if $x > r$, we can rewrite this formula as

$$\Pr(R = r) = \sum_{x>r}^{s} \frac{Pr(x)}{x}$$

Note that $\Pr(R = r) = \Pr(R = r - 1) - \frac{Pr(X=r)}{X=r}$ so that subtraction gives

$$\Pr(R = r - 1) - \Pr(R = r) = \frac{Pr(X = r)}{X = r}$$

Solving $Pr(X = r)$ results in

$$Pr(X = r) = (\Pr(R = r - 1) - \Pr(R = r)) \times r$$

From the probability mass fragment size distribution (est fragment size dist) we can calculate the complement of the cumulative distribution function $1 - F_X(x) = \Pr(X > x)$, which is the function needed for the coverage calculations. That is, $\Pr(X > x)$ gives us the proportion of fragments that can cover the CpG given that we observe a read starting at location x .

The expected contribution of a read to the coverage depends on the fragment size distribution in

the following way:

$$E(cov) = \sum_{x=0}^{s-1} \Pr(R = r)\Pr(X > r)$$

Thus, the expected coverage depends on both the read start distribution as well as the coverage distribution. Because the fragment size distribution is determined by the lab protocol and not directly related to the amount of methylation, we need to standardize on this expected contribution so that coverage estimates are not affected by inter-individual differences in fragment size distribution. The samples specific constant we need to multiply the coverage estimates with to standardize on fragment size distribution is the inverse of the expected read contribution:

$$\text{Coverage standardization factor sample } i = \frac{1}{E(\text{cov})_i}$$

2. Study samples

Adult C57BL/6 male mice (Jackson Laboratory, Bar Harbour, ME) were housed five per cage on a 12-h/12-h light/dark cycle in an AAALAC-accredited animal facility with continuous access to food and water. At 11-12 weeks of age the mice were anesthetized with 4% isoflurane followed by cardiac puncture. Brain tissue was extracted by a skilled technician, frozen in liquid nitrogen and stored in -80°C until DNA extraction. DNA was extracted using the Puregene kit (QIAGEN, Valencia, CA). All procedures were carried out in accordance with the “Guide for the Care and Use of Laboratory Animals” (Institute of Laboratory Animal Resources, National Academy Press, 1996) and were approved by the Institutional Animal Care and Use committee of Virginia Commonwealth University.

3. DNA methylation profiling

The samples were processed according to the standard protocols for SOLiD next-generation sequencing for paired-end barcoded fragment libraries (Life technologies, Foster City, CA). DNA samples were fragmented to a median size of about 150 bp through ultrasonication (Covaris, Woburn, MA). Size selection was conducted for the mouse samples to eliminate very short or long fragments. The methylated fraction of the genome, the methylome, was extracted using MethylMiner (Invitrogen, Carlsbad, CA) that uses the methyl-binding domain 2 (MBD2) protein for the capture. The captured DNA was eluted with 500 mM NaCl. This methylation enriched fraction of the genome was then used as input material for barcoded SOLiD next-generation sequencing fragment libraries. The library concentrations were measured using the SOLiD4 library Taqman quantitation kit and 7-8 barcoded samples were pooled in equal molarities prior to emulsion PCR. Automated emulsion PCR (ePCR) was conducted using

standard procedures for the SOLiD EZ bead system. The beads were deposited to the slides and sequenced on the SOLiD4 instruments using paired-end chemistry where 50 bp and 35 bp were read from each end of the fragments.

Sequence quality

The quality value (QV) for a particular color call is a function of its probability p that the color call is incorrect: QV = -10×log10(p). It takes two wrong adjacent color calls for a base call to change. Therefore, the quality value for the base call is approximately the sum of the QV for two adjacent color calls. The average QV was 23.6, meaning that the probability of an error occurring at a given color call is < 0.5%.

Alignment

The sequencing reads were aligned to the mouse reference genome (build 9/NCBI37) and human (build hg19/GRCh37) using Bioscope 1.2 (Life Technologies, Carlsbad, CA). Bioscope is a multithreaded application that aligns in color-space and takes full advantage of SOLiD's two-base encoding to increase the precision of the base calls. We first removed reads with more than 2 missing calls. We aligned QC'ed reads using a seed-and-extend approach with a seed of 25bp. Errors tend to increase towards the end of a read. Rather than considering the entire 25bp extension, we used local alignment to improve sensitivity by finding the maximum similarity score between the observed sequence and a substring of the reference sequence. For reads that could not be mapped, we used a second schema consisting of moving the start location of the seed from base 1 to base 15.

4. Simulation results

Figure S1 shows how the fragment size distribution type affected the precision in the simulation studies. The average absolute difference between estimated and real coverage functions is depicted for different curve types and sample sizes.

Figure S1. Simulation results for curve type

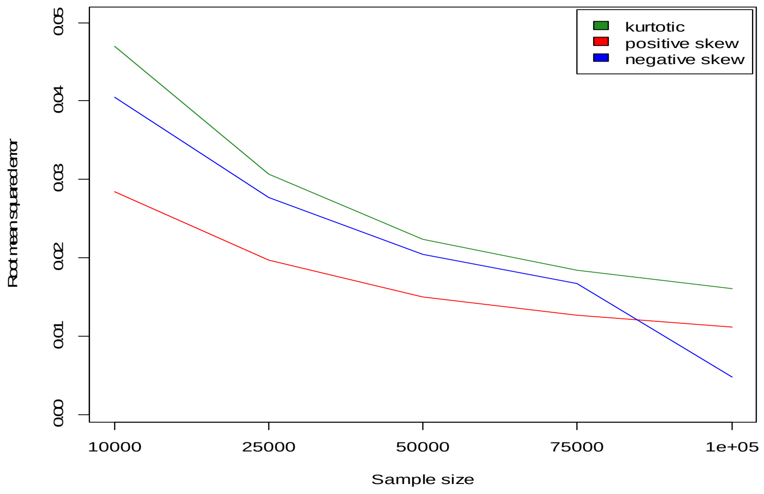

Figure S2 a-d displays estimated coverage functions for the conditions with 10,000, 25,000, 75,000, and 100,000 reads (results for the condition with 50,000 is shown in Figure 3 in the main text). Results are shown for the three fragment size distributions depicted in Figure 2 that were used to simulate the data, where the coverage function implied by these three distributions is depicted as well. The dashed lines indicate the mean of the estimated across all 10,000 simulations. The dotted line is an example of an estimated coverage function that has mean error identical to the 99th percentile of the 10,000 estimates.

Figure S2: Estimated coverage functions with 10k, 25k, 75k and 100k reads

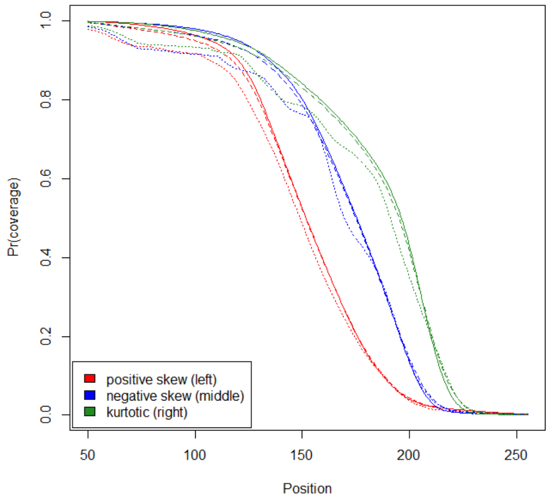

Figure S2a. Results for 10,000 reads

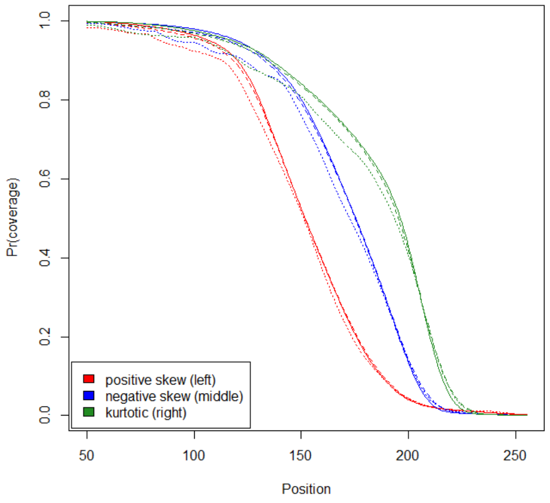

Figure S2b. Results for 25,000 reads

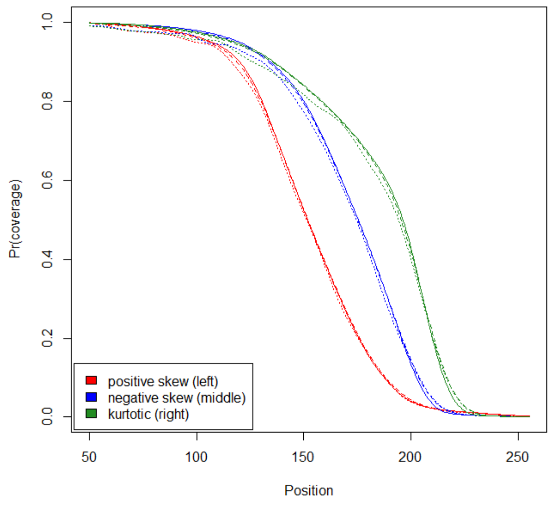

Figure S2c. Results for 75,000 reads

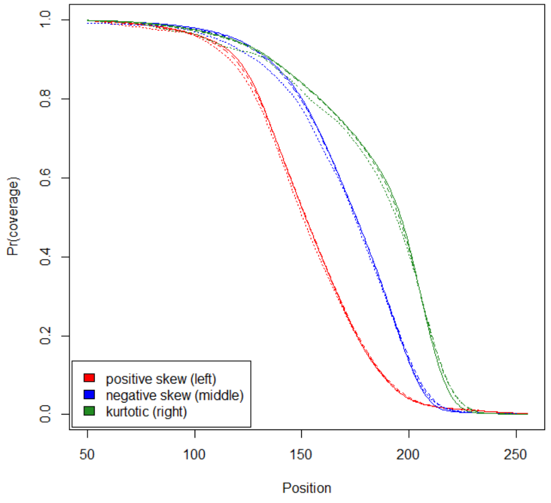

Figure S2d. Results for 100,000 reads

5. Plots of counts for read start positions

Figure S3 shows the plots with read start position distributions for each of the 8 samples. These distributions show systematic outliers at the very beginning of the read (positions 0-4). However, after these initial positions the frequencies of the read starts position do not show a systematic trend until the read length is reached. The decay after that point is expected and caused by parts of fragments becoming too short to cover the CpG (see Formula 1), which essentially forms the basis of our estimator. In other data we have sometimes observed a decay that starts before the read length is reached. However, this was the result of some fragments being shorter than the read length. Such fragments can end up in the data when the machine initially sequences into the adaptor but these reads are then subsequently “trimmed” during the alignment. Thus, when the methylated CpG is at the very beginning the read, the assumption of uniform read start distribution does not hold. However, as our estimator only uses the data starting from approximately the minimum read length, these outliers do not affect the estimation. The absence of a systematic trend until the minimum fragments length is reached suggests that the assumption of a uniform distribution for position-level read counts is reasonable for the range from which the data is used.

Figure S3. Counts of read start positions for each of the 8 samples.

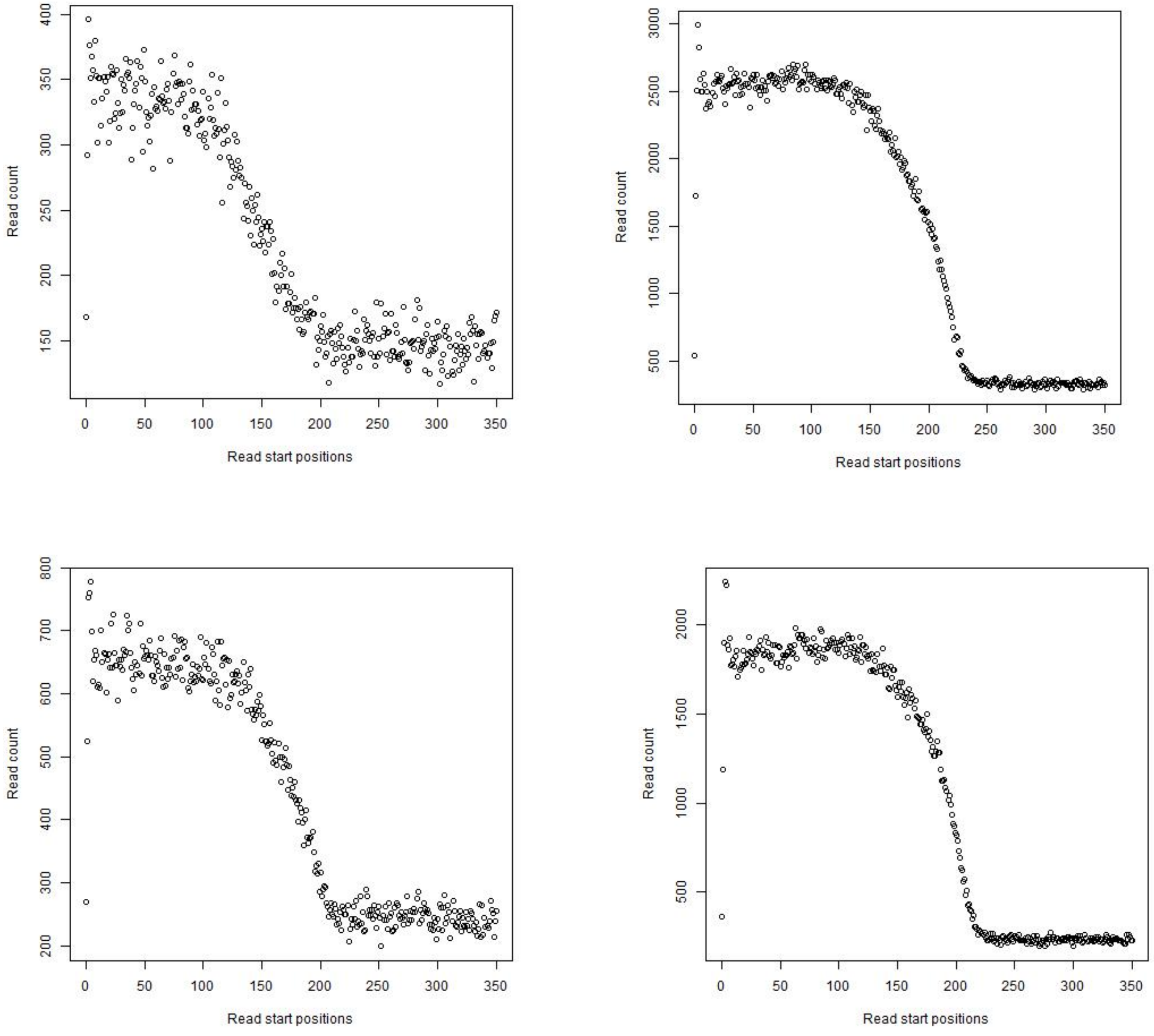

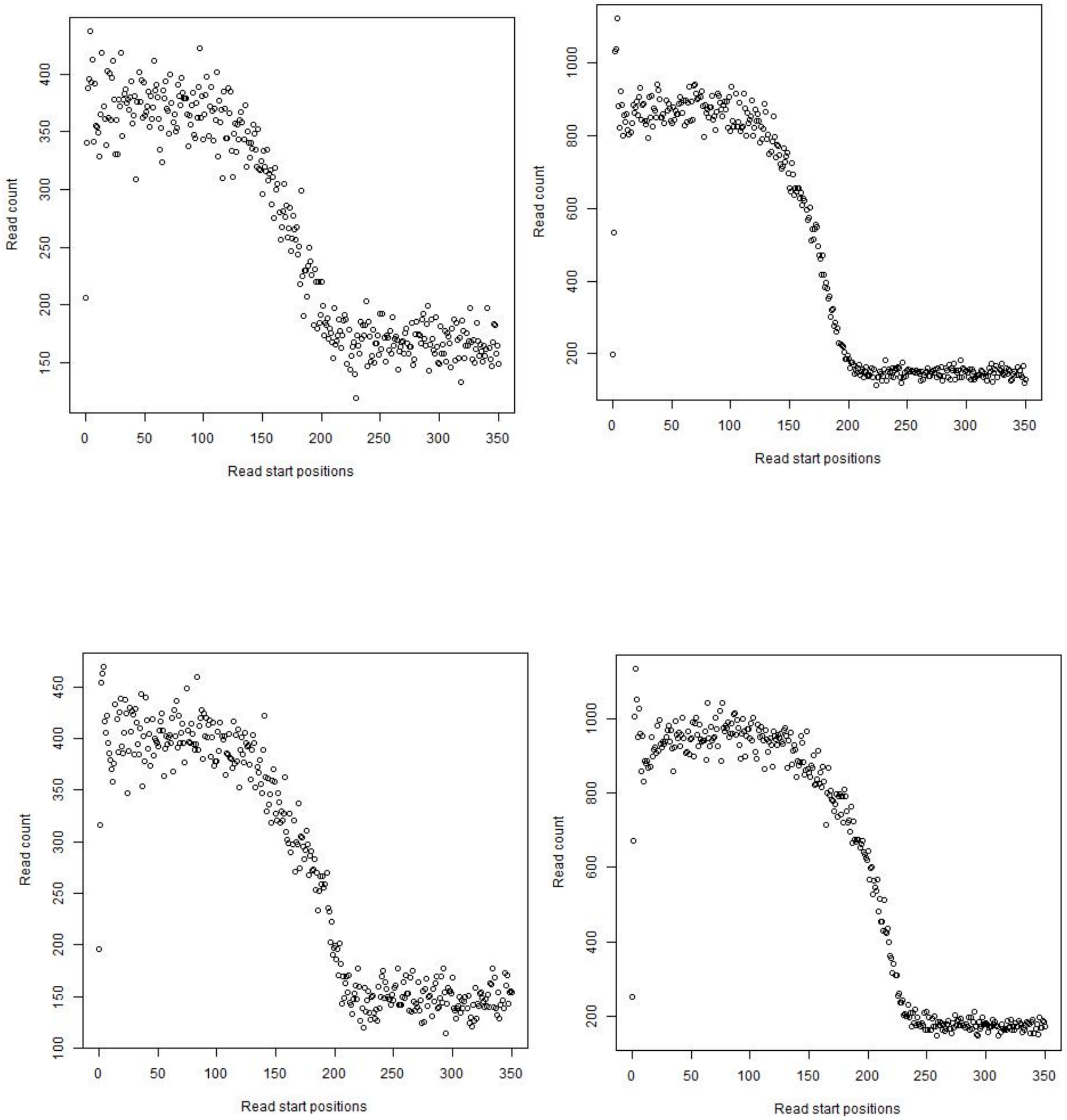
Read count
Read start positions
Read count
Read start positions
Read count
Read start positions
Read count
Read start positions
